# Comparative Profiling and In Silico Multitarget Analysis of Volatile Constituents from *Sambucus ebulus* L. Dried Fruits

**DOI:** 10.3390/plants15121765

**Published:** 2026-06-08

**Authors:** Stoyan Stoyanov, Ivayla Dincheva, Iliyan Kolev, Halil Şenol, Momchil Barbolov, Mladena Radeva, Paweł Olczyk, Petyo Boshnakov, Galina Yaneva, Diana Ivanova, Oskan Tasinov

**Affiliations:** 1 Department of Biology, Faculty of Pharmacy, Medical University of Varna, 84 Tzar Osvoboditel Blvd., 9002 Varna, Bulgaria; stoyan.stoyanov@mu-varna.bg (S.S.); galina.yaneva@mu-varna.bg (G.Y.); 2 Department of Agrobiotechnologies, AgroBioInstitute, Agricultural Academy, 8 Dr. Tsankov Blvd., 1164 Sofia, Bulgaria; ivadincheva@yahoo.com; 3 Department of Pharmaceutical Chemistry, Faculty of Pharmacy, Medical University of Varna, 84 Tzar Osvoboditel Blvd., 9002 Varna, Bulgaria; ilian.kolev@mu-varna.bg; 4 Department of Pharmaceutical Chemistry, Faculty of Pharmacy, Bezmialem Vakif University, Fatih, 34093 İstanbul, Türkiye; hsenol@bezmialem.edu.tr; 5 Department of Biochemistry, Molecular Medicine, and Nutrigenomics, Faculty of Pharmacy, Medical University of Varna, 84B Tzar Osvoboditel Blvd., 9002 Varna, Bulgaria; barbolov.m@gmail.com (M.B.); divanova@mu-varna.bg (D.I.); 6 Department of Ophthalmology and Visual Sciences, Faculty of Medicine, Medical University of Varna, 15 Doyran Street, 9000 Varna, Bulgaria; mladena.radeva@mu-varna.bg; 7 Faculty of Medical Sciences and Health Sciences, Radom University, Chrobrego 27, 26-600 Radom, Poland; p.olczyk@urad.edu.pl; 8 Department of International Economic Relations, University of Economics–Varna, 77 “Kniaz Boris I” Blvd., 9000 Varna, Bulgaria; ptboshnakov@yahoo.com

**Keywords:** dwarf elder, *S. ebulus*, volatile profile, molecular docking, molecular dynamics, MAO-A, COX-2, cholinesterase, neuroinflammation

## Abstract

Background: *Sambucus ebulus* L. berries contain numerous bioactive compounds, but their volatile molecular composition has not yet been fully characterized. In this study, we analyzed the volatile components of the essential oil and aqueous infusion prepared from dried *S. ebulus* fruits, followed by multitarget in silico analysis of the major compounds against the following enzymes implicated in neuroinflammation and oxidative stress, namely cyclooxygenase-2 (COX2), monoamine oxidases (MAO-A and MAO-B), acetylcholinesterase (AChE), and butyrylcholinesterase (BChE), for which inhibitory activity of plant-derived compounds has previously been reported. Methods: Steam distillation, gas chromatography-mass spectrometry (GC-MS), and gas chromatography-flame ionization detection (GC-FID) were employed for compound isolation and analysis. Molecular docking studies were performed using Schrödinger software (version 2025-1). Results: Fatty acid esters and hexahydrofarnesyl acetone predominated in the essential oil, whereas linalool was identified as the major constituent of the infusion. MAO-A was predicted to be the most favorable interaction target for hexahydrofarnesyl acetone and other major constituents. Conclusions: Our findings expand the currently limited available data on the volatile composition of *S. ebulus* fruits and characterize the volatile profile of its fruit infusion for the first time. The in silico analyses suggest that *S. ebulus* volatile constituents may interact with several target enzymes implicated in neurodegeneration and inflammation.

## 1. Introduction

**Figure 1:**
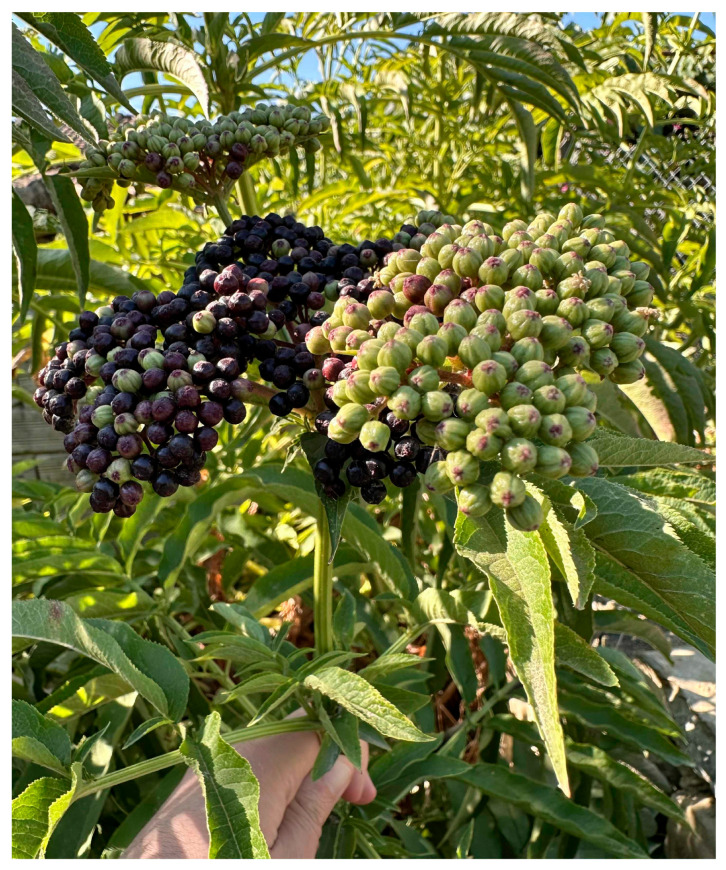
Ripe and unripe berries of *S. ebulus* (photograph by Stoyan Stoyanov). Immature fruits are green and poisonous due to the presence of the cyanogenic glycoside sambunigrin [8,9]. They usually ripen in August–September. Mature berries are dark blue or purple in color [1].

Several species of the genus *Sambucus* are widespread in Bulgaria, including *Sambucus nigra* L. (black elder), *Sambucus racemosa* L. (red-berried elder), and *Sambucus ebulus* L. (dwarf elder or danewort) [1]. Among these, *S. ebulus* has received comparatively less scientific attention despite its long-standing use in folk medicine across the Balkan Peninsula, Central Europe, Northwest Africa, and Southwest Asia [2]. Ripe berries are the most commonly utilized part of the dwarf elder (Figure 1) [2]. Their infusion is traditionally consumed for its purported immunomodulatory effects and for the relief of gastrointestinal inflammation [3,4].

At the molecular level, these ethnopharmacological effects may be associated with the reciprocal interplay between oxidative stress and inflammation [5]. Although the traditional use of *S. ebulus* fruit infusions has primarily been associated with gastrointestinal inflammation, growing evidence suggests that systemic inflammation and oxidative stress are closely linked to neuroinflammatory processes through the gut–brain axis [6]. Chronic peripheral inflammation can trigger microglial activation, promote the release of pro-inflammatory mediators, and contribute to neuronal dysfunction [7]. Therefore, phytochemicals capable of modulating oxidative stress and inflammation at the systemic level may exert both direct and indirect neuroprotective effects. In this context, investigating the bioactive potential of *S. ebulus*, particularly its less explored volatile constituents, within a neuroinflammation-related framework, represents a rational extension of its traditional use.

Phytochemical studies on dwarf elder have focused primarily on non-volatile constituents, particularly polyphenols such as hydroxycinnamic acids, flavonols, anthocyanins, condensed tannins, and stilbenes [4,10,11,12]. These compounds are considered major contributors to the plant’s antioxidant and anti-inflammatory properties [4,13]. Their biological activities have been confirmed through in vitro experiments, animal models, and clinical trials, demonstrating beneficial effects on oxidative stress, inflammatory mediator activity, and endoplasmic reticulum stress [4,12,14,15,16,17].

Essential oils, which are complex mixtures of volatile organic compounds (VOCs), have attracted increasing interest due to their diverse biological activities, including antiviral, anti-inflammatory, antimicrobial, antioxidant, neuroprotective, and anticancer effects [18,19,20,21,22]. In recent years, particular attention has been given to the synergistic antioxidant effects of different essential oils [23]. In contrast to phenolic compounds, VOCs in *S. ebulus* have received considerably less attention. The flowers of *S. ebulus* have been reported to contain up to 0.3% essential oil, composed predominantly of free fatty acids and alkanes [24]. A study conducted in 2010 identified 60 VOCs in elderberry leaves, with β-bisabolene (11.4%), germacrene D (6.9%), geranyl acetate (5.6%), and cubebene (5.2%) being the most abundant [24]. Other studies have also identified terpene-dominated VOC profiles in leaves, demonstrating that plant growth regulators can substantially alter the relative composition of the essential oil by enhancing or suppressing individual constituents [25,26]. The essential oil yield from the berries has been reported to be approximately 0.01% [9,24], containing eugenol, valeric acid, methyl salicylate, hexanol, methyl palmitate, methyl linoleate, among others [27]. To the best of our knowledge, the presence of VOCs in infusions prepared from the berries has not been investigated.

Chronic inflammation and oxidative stress contribute to neuronal damage, synaptic dysfunction, and progressive cognitive decline in Alzheimer’s disease [28,29]. During neuroinflammation, glial cells become activated, inflammatory mediators are released, oxidative stress is amplified, and blood–brain barrier dysfunction occurs, collectively contributing to neuronal injury [30]. Research highlights the interconnected role of several enzymes in these pathological mechanisms associated with Alzheimer’s disease [31]. Neuroinflammation leads to the overproduction of cyclooxygenase-2 (COX-2), whose excessive activity exacerbates oxidative stress and neuronal damage, making it an important target for anti-inflammatory intervention [32,33]. Monoamine oxidases, particularly MAO-A and MAO-B, play crucial roles in the metabolism of neurotransmitters such as dopamine, serotonin, and norepinephrine. Elevated MAO activity generates reactive oxygen species (ROS), thereby contributing to oxidative stress, mitochondrial dysfunction, and neuronal death, ultimately accelerating neurodegenerative processes [34]. Meanwhile, acetylcholinesterase (AChE) and butyrylcholinesterase (BChE) regulate acetylcholine levels, which are essential for memory formation and cognitive function. In Alzheimer’s disease, increased cholinesterase activity leads to accelerated acetylcholine depletion, thereby worsening cognitive impairment and synaptic dysfunction [35,36].

Molecular docking and molecular dynamics simulations are in silico tools that enable the rapid prediction of ligand-protein interactions while providing mechanistic insights into potential biological effects [37]. The selection of COX-2, MAO-A, MAO-B, AChE, and BChE as molecular targets is justified by their complementary roles in neuroinflammation, oxidative stress, and neurotransmitter regulation. These enzymes are established pharmacological targets for low-molecular-weight lipophilic compounds, including volatile phytochemicals. Due to their small size and hydrophobic nature, VOCs may readily interact with enzyme active sites and potentially cross the blood–brain barrier [38]. Thus, investigating VOC–enzyme interactions provides a mechanistically relevant framework for evaluating their multitarget neuroprotective potential [39,40,41].

In recent years, numerous studies have demonstrated that both synthetic and plant-derived compounds can act as potent inhibitors of these enzymes, showing promising effects on disease-related pathways [42,43,44]. For instance, tryptamine derivatives have been shown to inhibit AChE, MAO-B, and COX-2 in vitro, with molecular docking analyses supporting their proposed binding modes [31]. Similarly, clozapine has been identified as a dual AChE and COX-2 inhibitor through docking studies, while in vivo evidence suggests neuroprotective effects mediated through the modulation of inflammatory markers [45]. In addition, natural compounds such as ursolic acid and rosmarinic acid from *Rosmarinus officinalis* have demonstrated binding affinities toward AChE comparable to those of Donepezil, further highlighting their therapeutic potential [46]. Volatile organic compounds, particularly terpenes, phenylpropanoids, and fatty acid derivatives, are known to possess significant biological activities relevant to neurodegenerative disorders. Several VOCs, including eugenol and various terpenoid derivatives, have been reported to exhibit inhibitory activity against key enzymes such as AChE, monoamine oxidases, and COX-2, in addition to antioxidant and anti-inflammatory effects [47,48,49]. These findings suggest that VOCs may represent more than minor accompanying phytochemicals and could act as bioactive agents capable of physiologically relevant modulation. Despite these promising observations, the contribution of VOCs from *S. ebulus* remains largely unexplored within a multitarget pharmacological framework. Therefore, further studies are needed to fully establish the biological relevance and therapeutic potential of volatile phytochemicals in this context.

The present study aimed to perform a comparative qualitative and quantitative analysis of VOCs present in both the essential oil and aqueous infusion of dried *S. ebulus* fruits. In addition, the major volatile constituents were evaluated through a multitarget in silico approach against COX-2, MAO-A, MAO-B, AChE, and BChE. This strategy was designed to assess whether VOCs, as structurally distinct and relatively underexplored phytochemicals, may contribute to the modulation of interconnected pathways linking oxidative stress, inflammation, and neuroinflammation, thereby providing a mechanistic rationale connecting the traditional use of *S. ebulus* with its potential modern relevance to neurodegenerative disorders.

## 2. Materials and Methods

### 2.1. Plant Material

The plant material was purchased from a local market (Neven, Varna, Bulgaria) and supplied by the manufacturer Bilki EOOD (Sinitovo Village, Industrial Zone, Bulgaria). The material was collected in September from the Eastern Rhodopes region (approximate coordinates: 41.6° N, 25.4° E). After harvesting, the fruits were dried in a drying oven (Memmert UF110, Schwabach, Germany) at 50–60 °C. For botanical verification and traceability, a voucher specimen of *S. ebulus* fruits was deposited under No. 108144 in Herbarium SO (Index Herbariorum), Faculty of Biology, Sofia University St. Kliment Ohridski.

The infusion preparation protocol was inspired by Bulgarian traditional medicine and adapted based on previously published research [13]. In summary, 2.5 g of dried berries were infused with 300 mL of distilled water heated to 100 °C, followed by incubation for 30 min in a closed glass vessel. No filtration was applied.

### 2.2. Extraction of Volatile Compounds

Volatile constituents (essential oil fraction) were extracted from dried *S. ebulus* berries by steam distillation using a laboratory-scale distillation apparatus with a capacity of 0.01 m^3^. The steam generator was fabricated from stainless steel, whereas the remaining apparatus components, including the distillation vessel and condensers, were made of borosilicate glass. An autoclavable amber borosilicate glass bottle was used as the receiver. The distillation vessel was loaded with 1.5 kg of berries. The productivity of the configured distillation system was estimated at 1.1 L/h. The resulting hydrolate (approximately 2.5 L) was subjected to two consecutive liquid–liquid extraction steps using pentane (total volume: 20 mL) to isolate the essential oil fraction. The combined organic phases were concentrated in vacuo under a gentle argon stream (5 mL/min) using a programmable rotary vacuum evaporator coupled to a mass flow controller.

For the isolation of VOCs from the prepared infusion, an analogous extraction procedure was applied. After cooling to room temperature, the entire infusion volume (300 mL) was extracted twice with pentane (total volume: 20 mL). Due to the trace essential oil yield (microgram quantities), filtration of neither the infusion nor the VOC-enriched pentane extract was required. Instead, the organic phase was carefully transferred using a glass pipette into a 25 mL round-bottom flask. Concentration of the resulting extract was performed as described above.

Subsequently, both sample types were transferred into amber glass chromatographic vials and stored under light-protected conditions prior to chromatographic analysis.

### 2.3. Analysis of Volatile Compounds

Analytical repeatability was assessed by analyzing essential oil and infusion samples in technical replicates (*n* = 2 and *n* = 4, respectively). The chemical profiles were determined using an Agilent Technologies 7890A gas chromatograph coupled to a 5975C mass-selective detector (Santa Clara, CA, USA). Separation was achieved on an HP-5ms fused-silica capillary column (30 m × 0.32 mm i.d., 0.25 μm film thickness). The GC oven temperature program was as follows: initial temperature 60 °C (held for 3 min), increased to 80 °C at 1 °C·min^−1^ and held for 3 min, followed by an increase to 300 °C at 5 °C·min^−1^ with a final hold of 5 min. Helium was used as the carrier gas at a flow rate of 1.0 mL·min^−1^. A 1.0 μL aliquot was injected in split mode (20:1). The injector, ion source, and quadrupole temperatures were maintained at 250 °C, 230 °C, and 150 °C, respectively. Mass spectra were acquired in full-scan electron ionization (EI) mode at 70 eV. Compound identification was based on comparison of mass spectral fragmentation patterns with the NIST 08 and Adams libraries, combined with linear retention index (LRI) data. LRIs were calculated relative to a homologous series of n-alkanes (C8–C36) analyzed under identical chromatographic conditions and compared with literature values. Tentative identification was accepted when the mass spectral similarity index was ≥80% and the experimentally determined LRI differed from reference values by no more than ±10 index units. As no authentic reference standards were used, all compound identifications should be regarded as tentative.

Quantitative analysis was performed using GC-FID on the same chromatographic system and column under the temperature program described above. The injector and detector temperatures were set at 220 °C and 280 °C, respectively. Helium served as the carrier gas at 1.0 mL·min^−1^, and 1.0 μL of each sample was injected in split mode. The relative percentage of each VOC component was calculated as a percentage of the total FID area using the peak normalization method for both essential oil and infusion samples.

### 2.4. Computational Studies

Molecular docking and molecular dynamics simulations were performed using Schrödinger Molecular Modeling Suite (version 2025-1), including the Maestro interface (version 14.3) and Desmond (D. E. Shaw Research 2024-4). Target proteins included COX-2 (PDB ID: 4PH9; 1.81 Å), MAO-A (PDB ID: 2Z5X; 2.20 Å), MAO-B (PDB ID: 4A79; 1.89 Å), AChE (PDB ID: 4EY7; 2.35 Å), and BChE (PDB ID: 6EP4; 2.30 Å), retrieved from the Protein Data Bank. Protein structures were prepared using the Protein Preparation Wizard, including the addition of missing hydrogen atoms, optimization of protonation states, and energy minimization to remove steric clashes. Ligands were prepared using the LigPrep module, generating all relevant ionization and tautomeric states at pH 7.4 ± 2, followed by geometry optimization and energy minimization using the OPLS4 force field. Docking was performed using Glide and Induced Fit Docking (IFD). For Glide docking, a cubic grid box (20 × 20 × 20 Å) was centered on the active site to fully encompass the binding pocket. The best docking poses were selected based on IFD scores, and binding free energies were estimated using Prime MM-GBSA with the VSGB solvation model to account for both polar and hydrophobic contributions [36,50].

To assess dynamic stability, 250 ns molecular dynamics simulations were carried out using Desmond. Each complex was solvated in a TIP4P water box containing Na^+^ and Cl^−^ ions to neutralize the system. Following energy minimization, systems were equilibrated in the NPT ensemble at 300 K and 1 atm, followed by 250 ns production runs. Stability analyses included RMSD of protein Cα and ligand atoms, RMSF of residues and ligand atoms, and monitoring of hydrogen bonds, hydrophobic contacts, and other key intermolecular interactions throughout the simulation [51].

### 2.5. Statistical Analysis

Statistical analysis was performed using GraphPad Prism v7.0 (GraphPad Software, La Jolla, CA, USA). The study design included technical replicates (*n* = 2 for essential oil; *n* = 4 for infusion) derived from a single biological sample per group (biological *n* = 1). Therefore, the dataset does not meet the assumptions required for inferential statistical analysis, including independence and estimation of biological variability. Accordingly, no hypothesis testing, *p*-values, or normality assessments (e.g., Shapiro–Wilk test) were performed, as such analyses would not be statistically valid or interpretable. Data are presented as mean ± standard deviation (SD) to reflect analytical repeatability (instrumental and procedural variability) rather than biological variation.

## 3. Results

### 3.1. Qualitative and Quantitative Analysis of the Essential Oil and Infusion

**Figure 2:**
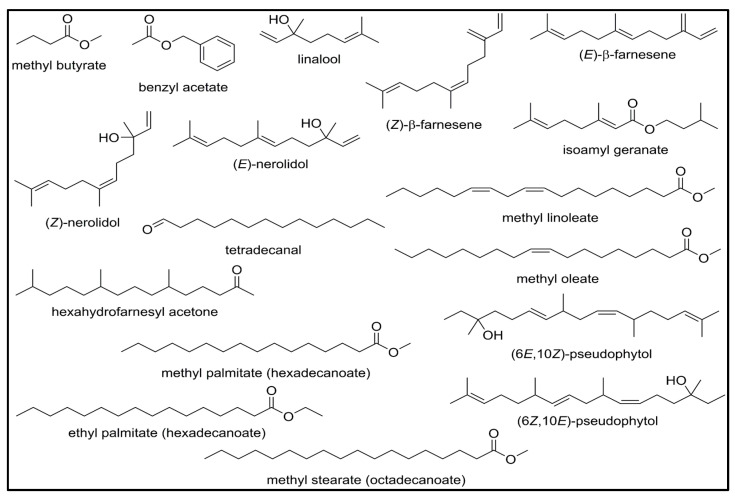
Chemical structure of methyl butanoate (methyl butyrate), benzyl acetate, linalool, (E)-β-farnesene, (Z)-nerolidol, (E)-nerolidol, tetradecanal, isoamyl geranate, hexahydrofarnesyl acetone, methyl hexadecanoate (methyl palmitate), ethyl hexadecanoate, (6E,10Z)-pseudophytol, (6Z,10E)-pseudophytol, methyl linoleate, methyl oleate, methyl octadecanoate (methyl stearate). Chemical structures were generated by Iliyan Kolev using ChemBioOffice Ultra 9 software (2026) (accessed on 17 February 2026).

**Figure 3:**
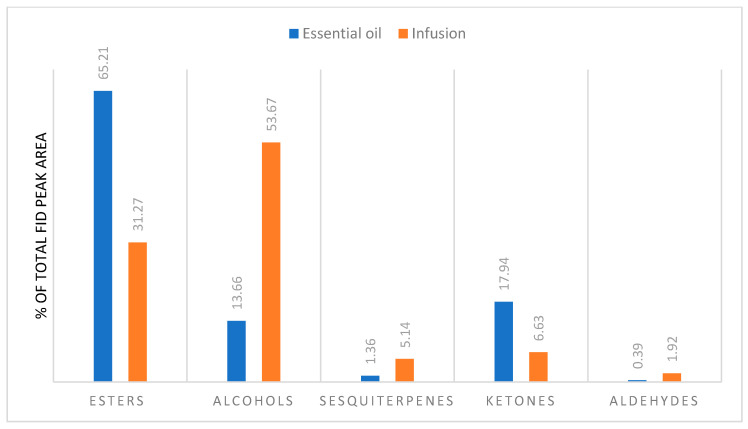
Relative distribution of the main classes of VOCs (% of total FID peak area) identified in the essential oil and aqueous infusion prepared from dried *S. ebulus* fruits.

In total, seventeen volatile compounds were identified, most of which were detected in both the essential oil and the fruit infusion (Table 1). They included eight esters, one aldehyde, one ketone, two sesquiterpenes, and five alcohols (Figure 2). As shown in Figure 3, the essential oil was dominated by fatty acid esters (65.21%), followed by ketones (17.94%) and alcohols (13.66%). In contrast, the infusion exhibited a predominance of alcohols (53.67%), with esters (31.27%) and ketones (6.63%) also contributing substantially. Minor constituents identified in both sample types included sesquiterpenes and aldehydes. These differences indicate a marked shift in volatile class distribution between the two preparations. Major constituents were defined as compounds present at levels exceeding 5% of the total FID peak area. Representative total ion chromatograms for both sample types are presented in Figure S1.

A total of sixteen VOCs were identified in the essential oil. Quantitative analysis revealed that methyl hexadecanoate exhibited the highest relative proportion (23.97 ± 2.59), followed by hexahydrofarnesyl acetone (17.94 ± 1.65), methyl oleate (15.61 ± 1.12), methyl linoleate (13.38 ± 1.24), and linalool (6.41 ± 1.39). The remaining compounds were present in trace amounts.

Fifteen VOCs were identified in the infusion. Comparison between the two matrices revealed notable differences in their relative volatile composition. The infusion was characterized by a predominance of linalool (51.29 ± 2.60%), which exhibited a substantially higher relative proportion than in the essential oil (6.41 ± 1.39%). Similarly, methyl butanoate was present at a higher relative proportion in the infusion (11.83 ± 1.59%) compared with the essential oil (3.96 ± 0.95%). Benzyl acetate was detected exclusively in the infusion (9.72 ± 1.17%) and was absent from essential oil samples. Hexahydrofarnesyl acetone (6.63 ± 2.00%) was also identified as a major constituent. In addition, *n*-tetradecanal, (Z)-β-farnesene, and (E)-β-farnesene exhibited higher relative proportions in the infusion, whereas (Z)-nerolidol and (E)-nerolidol were not detected.

### 3.2. Molecular Docking Studies

Molecular docking analyses were performed to investigate the interactions of the most abundant phytochemical constituents identified in *S. ebulus* extracts. The selected compounds—benzyl acetate, hexahydrofarnesyl acetone, linalool, methyl butanoate, methyl hexadecanoate, methyl linoleate, and methyl oleate—were docked into the active sites of MAO-A, MAO-B, AChE, BChE, and COX-2 to evaluate their potential binding interactions.

The selection of multiple biological targets was guided by both the ethnopharmacological use of *S. ebulus* and the interconnected nature of the underlying pathological processes. Traditionally, *S. ebulus* has been used for the management of inflammatory conditions, which are closely associated with oxidative stress and, in some cases, neuroinflammatory pathways. Enzymes such as COX-2, MAO-A, MAO-B, AChE, and BChE are functionally interconnected within biological networks involving inflammation, redox imbalance, and neurotransmitter regulation. Accordingly, a multitarget approach was adopted to better reflect the potential polypharmacological properties of volatile phytochemicals and to explore their possible interactions across these interrelated pathways.

The docking results, including IFD Glide scores and MM-GBSA ΔG binding energies, are summarized in Table 2. Induced Fit Docking (IFD) was performed to account for the conformational flexibility of both ligands and key amino acid residues within the active site, allowing side-chain and limited backbone adjustments upon ligand binding. This procedure generated optimized ligand–protein complexes for subsequent analysis. MM-GBSA calculations were then applied as a post-docking approach to estimate binding free energies based on these refined conformations. Unlike IFD, MM-GBSA does not involve extensive conformational sampling during pose generation but instead evaluates the energetic stability of the predicted binding conformations, thereby improving confidence in the estimated binding affinities.

Among the investigated compounds, hexahydrofarnesyl acetone exhibited the most favorable predicted binding affinity toward MAO-A (IFD Glide score: −10.04 kcal/mol; MM-GBSA ΔG: −67.88 kcal/mol), suggesting a stable binding conformation within the active site. This compound also demonstrated predicted interactions with MAO-B, COX-2, and AChE. However, these findings are based exclusively on computational predictions and should not be interpreted as evidence of superior inhibitory activity.

Smaller molecules such as benzyl acetate and linalool exhibited weaker but still notable predicted binding, particularly toward MAO-A and AChE, suggesting possible supportive contributions in combination with higher-affinity ligands. Overall, the docking results indicate that MAO-A may represent a relevant interaction site for several of the investigated VOCs, whereas COX-2, MAO-B, and the cholinesterases may constitute secondary targets. Nevertheless, these findings should be interpreted solely as indicators of potential binding tendencies within a computational framework. Further in vitro and in vivo studies are required to establish their biological relevance.

The ligand–protein interaction (LPI) profiles of all investigated compounds were further analyzed to elucidate their binding modes, key interaction sites, and stabilization mechanisms, with particular emphasis on MAO-A.

Several VOCs yielded docking scores and MM-GBSA binding energies within a comparable numerical range for certain targets. However, such observations should be interpreted cautiously, as docking scores and binding energy estimates do not directly correspond to biochemical potency, pharmacological efficacy, or therapeutic equivalence. Accordingly, the present findings should be regarded as preliminary in silico evidence of possible interactions rather than definitive proof of inhibitory activity.

It should also be noted that molecular docking and MM-GBSA analyses provide theoretical estimations of ligand–protein interactions and do not account for factors such as bioavailability, metabolism, or experimentally determined enzyme inhibition, all of which are critical for assessing actual pharmacological activity.

**Figure 4:**
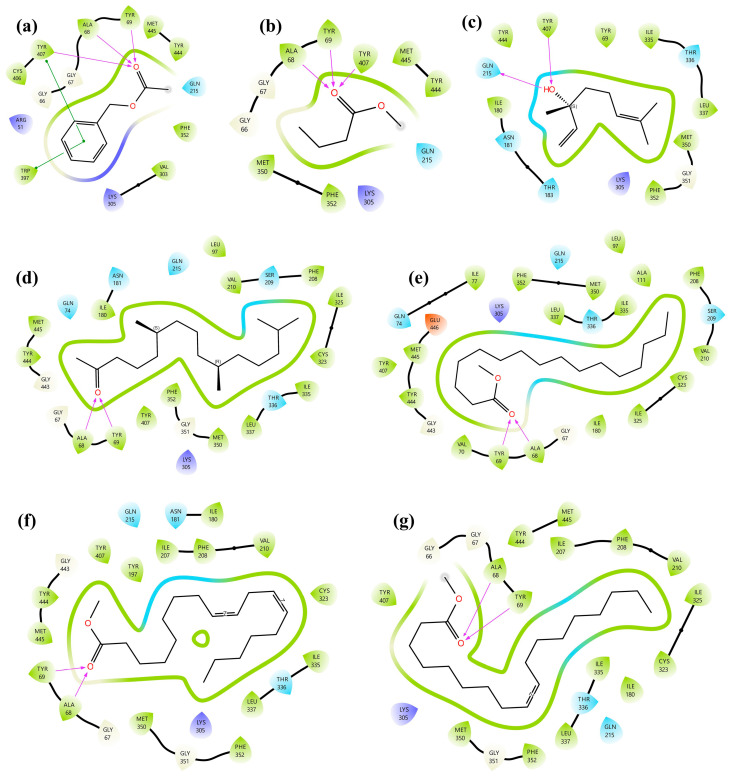
Molecular docking 2D LPI of titular compounds against MAO-A. (**a**) Benzyl acetate, (**b**) methyl butanoate, (**c**) linalool, (**d**) hexahydrofarnesyl acetone, (**e**) methyl hexadecanoate, (**f**) methyl linoleate and (**g**) methyl oleate.

The 2D ligand–protein interaction representations of the investigated compounds in complex with MAO-A are presented in Figure 4.

The 2D interaction analysis revealed both polar and nonpolar contributions to ligand binding, highlighting several residues involved in complex stabilization. Benzyl acetate forms three hydrogen bonds through its ester carbonyl with Tyr-407, Ala-68, and Tyr-69, while its aromatic ring engages in π–π stacking interactions with Tyr-407 and Trp-397, suggesting a dual polar–aromatic anchoring mechanism (Figure 4a). Methyl butanoate forms three hydrogen bonds through its ester carbonyl with Ala-68, Tyr-69, and Tyr-407, indicating consistent polar stabilization (Figure 4b). Linalool establishes two hydrogen bonds via its tertiary alcohol group with Gln-215 and Tyr-407, demonstrating the stabilizing contribution of polar hydroxyl interactions within the MAO-A active site (Figure 4c).

Hexahydrofarnesyl acetone forms two hydrogen bonds through its ketone carbonyl with Ala-68 and Tyr-69, while its extended hydrophobic chain is stabilized by multiple nonpolar contacts, emphasizing substantial van der Waals contributions (Figure 4d). Similarly, the long-chain esters methyl hexadecanoate, methyl linoleate, and methyl oleate each form two hydrogen bonds via their ester carbonyl groups with Ala-68 and Tyr-69, further emphasizing these residues as conserved anchoring points across multiple ligands (Figure 4e–g). Overall, Ala-68, Tyr-69, Tyr-407, Gln-215, and Trp-397 emerged as the principal residues mediating hydrogen bonding and π–π interactions, while hydrophobic contacts surrounding long aliphatic chains provided additional stabilization. These findings suggest a partially shared mode of MAO-A engagement among the major volatile constituents of the extract.

### 3.3. Molecular Dynamics Simulations

**Figure 5:**
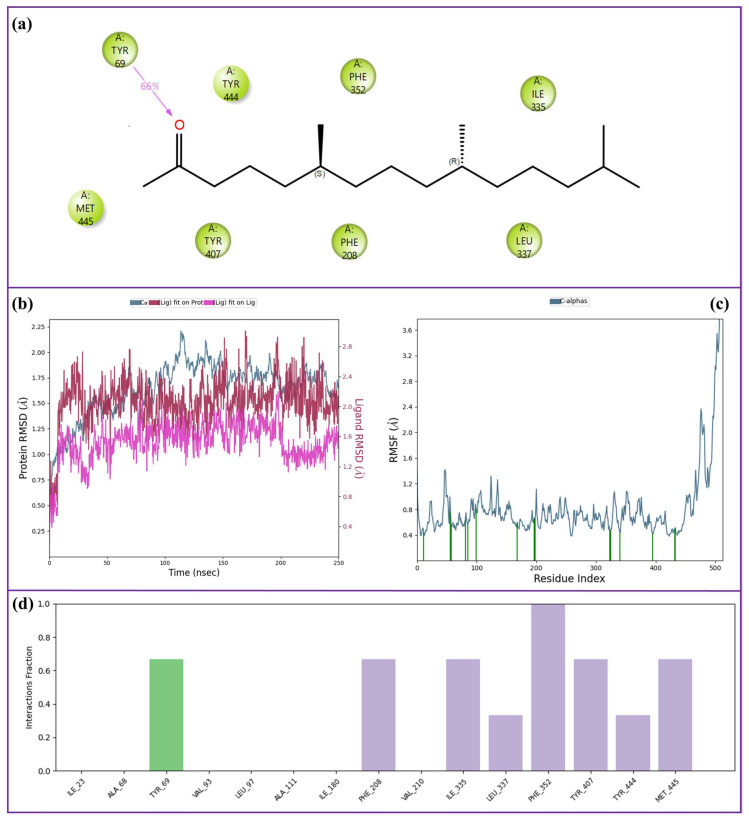
MD simulation analysis of Hexahydrofarnesylacetone–MAO-A complex. (**a**) 2D key ligand protein interactions, (**b**) RMSD of ligand and protein atoms, (**c**) RMSF of protein atoms and (**d**) fractional interaction histogram.

The hexahydrofarnesyl acetone–MAO-A complex was selected for molecular dynamics (MD) simulation based on its favorable docking score and MM-GBSA binding energy among the investigated compounds, as well as its consistent interaction profile within the MAO-A active site. A 250 ns MD simulation was performed to evaluate the stability and dynamic behavior of this representative complex. RMSD and RMSF analyses were used to assess protein backbone stability and residue flexibility, while interaction histograms were employed to monitor hydrogen bonding and hydrophobic contacts over time. The results are presented in Figure 5.

As shown in Figure 5a, Tyr-69 emerged as a key anchoring residue, forming a hydrogen bond with the ligand for approximately 66% of the simulation time, while the hydrophobic tail of hexahydrofarnesyl acetone remained surrounded by nonpolar residues throughout the simulation. RMSD analysis (Figure 5b) indicated that the protein Cα backbone remained stable, with an average RMSD of 1.75 Å. The ligand RMSD values (approximately 2.0 Å when fitted to the protein and 1.6 Å when fitted to itself) suggest moderate conformational adaptation within the binding pocket. RMSF values (Figure 5c) averaged around 1.0 Å for Cα atoms, indicating limited flexibility, particularly in the active site region. Fractional interaction analysis (Figure 5d) identified Tyr-69, Phe-208, Ile-335, Leu-337, Phe-325, Tyr-444, and Met-445 as residues contributing to ligand stabilization through hydrogen bonding and hydrophobic interactions.

It should be noted that only one representative complex was subjected to MD simulation in this study. Therefore, the results provide a case-specific illustration of the dynamic behavior of hexahydrofarnesyl acetone within the MAO-A binding site and should not be generalized to all compounds or targets. Further MD simulations of additional ligand–protein complexes would be necessary to establish broader conclusions.

### 3.4. Molecular Docking Validations

**Figure 6:**
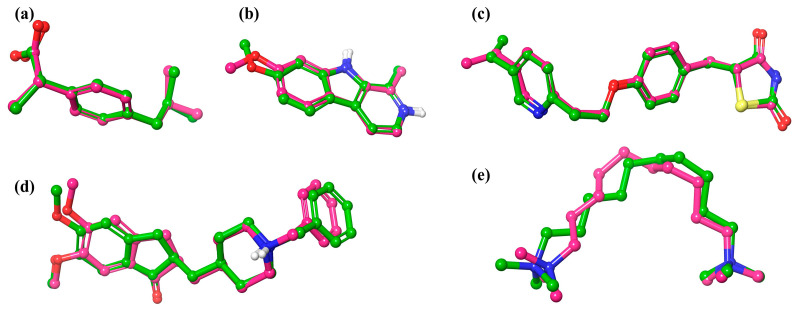
Docking protocol validation by redocking of co-crystallized ligands into target proteins. The native co-crystallized ligands are represented by a green skeleton, while the redocked ligand poses are represented by a pink skeleton. Atom colors are shown as follows: oxygen (red), nitrogen (blue), and sulfur (yellow). (**a**) COX-2 (4PH9, ibuprofen, RMSD = 0.80 Å); (**b**) MAO-A (2Z5X, harmine, RMSD = 0.47 Å); (**c**) MAO-B (4A79, pioglitazone, RMSD = 0.52 Å); (**d**) AChE (4EY7, donepezil, RMSD = 0.46 Å); (**e**) BChE (6EP4, decamethonium, RMSD = 0.72 Å).

Docking validation was performed for all target proteins by redocking their respective co-crystallized ligands into the defined active sites: COX-2 (PDB ID: 4PH9; resolution: 1.81 Å; co-crystallized ligand: ibuprofen), MAO-A (PDB ID: 2Z5X; resolution: 2.20 Å; co-crystallized ligand: harmine), MAO-B (PDB ID: 4A79; resolution: 1.89 Å; co-crystallized ligand: pioglitazone), AChE (PDB ID: 4EY7; resolution: 2.35 Å; co-crystallized ligand: donepezil), and BChE (PDB ID: 6EP4; resolution: 2.30 Å; co-crystallized ligand: decamethonium). The co-crystallized ligands (green) and their corresponding redocked poses (pink) were superimposed to evaluate the accuracy of the docking protocol (Figure 6). The calculated RMSD values were 0.80 Å for COX-2 (4PH9), 0.47 Å for MAO-A (2Z5X), 0.52 Å for MAO-B (4A79), 0.46 Å for AChE (4EY7), and 0.72 Å for BChE (6EP4). The close overlap between experimental and predicted binding modes, together with all RMSD values well below the accepted threshold of 2.0 Å, confirms that the docking protocol is robust and reliable for predicting ligand–protein interactions in this study.

## 4. Discussion

Our phytochemical analysis adds to the currently available information on the composition of *S. ebulus* fruits, with a particular focus on volatile compounds.

A comparison with previously published data reveals notable differences in the dominant volatile constituents of *S. ebulus* fruits. Earlier studies have reported eugenol and valeric acid as the major components [27], whereas the present study identified various esters as the predominant constituents. These differences may reflect chemotypic variability within the species, potentially influenced by genetic and environmental factors [52]. In comparison, the volatile composition of *S. nigra* fruits is mainly characterized by oxygenated monoterpenes, while data on *S. racemosa* remain limited [52,53].

The extraction method influences both the qualitative and quantitative composition of VOC profiles. Distillation tends to favor the recovery of lipophilic and volatile compounds that partition into the oil fraction [54,55]. Accordingly, the essential oil profile was dominated by fatty acid esters, followed by hexahydrofarnesyl acetone. Certain sesquiterpene alcohols, such as nerolidol, were detected only in the oil fraction, likely reflecting their hydrophobic nature and affinity for non-polar matrices [56]. Although present in low concentration, β-farnesene, a highly lipophilic sesquiterpene, was found in greater relative proportion in the infusion [57]. This observation may be related to methodological factors affecting compound recovery during steam distillation, as previously suggested by Gawde et al. (2014) [58]. *N*-tetradecanal, a long-chain aldehyde with low water solubility, was more prominent in the infusion [59]. Although the mechanism underlying this observation was not investigated here, interactions with other fruit constituents, including flavonoids known to occur abundantly in elderberries, could hypothetically influence its distribution [60,61]. A similar consideration may apply to isoamyl geranate.

The infusion exhibited a higher relative proportion of polar and water-soluble compounds [62]. Methyl butanoate is also a fatty acid ester, but it is a small, moderately polar molecule [63]. For this reason, it likely predominates in the infusion. We do not exclude the possibility that it migrates into the aqueous fraction of the distillate [64]. Likewise, linalool, as a short-chain terpene alcohol with a hydroxyl group, may display partial affinity for the aqueous phase [65]. One of the main components in the water extract, benzyl acetate, was found only in the infusion, although it is practically insoluble in water [66]. Its absence from the oil fraction may hypothetically reflect partial transformation during heating, although this was not experimentally examined in the present study [54,67]. It should also be noted that no filtration was applied during the preparation of the infusion. The presence of suspended particulate matter may have influenced the extraction and distribution of volatile compounds and this potential effect was not investigated in the present study, so it should be taken into account when interpreting the results.

The differing chemical compositions of the extracts may contribute to distinct biological properties. The main components in the essential oil included methyl esters of fatty acids, hexahydrofarnesyl acetone, and linalool. Among these, linalool is widely reported to exhibit antioxidant, anti-inflammatory, analgesic, antibacterial, antiparasitic, anticancer and other activities in various experimental models [68,69,70,71,72,73,74,75,76,77,78]. Methyl hexadecanoate and ethyl hexadecanoate have been associated with anti-inflammatory, vasodilatory, and antihyperglycemic effects [79,80,81,82,83]. Hexahydrofarnesyl acetone has been linked to antimicrobial, antinociceptive, antioxidant, and anti-inflammatory activities [84,85,86]. Methyl linoleate and methyl oleate have also demonstrated diverse biological effects in previous studies [87,88,89,90,91]. However, these findings derive from studies on isolated compounds or other experimental systems and should not be interpreted as direct evidence of comparable activity for the *S. ebulus* extracts analyzed here. Further experimental studies are required to determine whether these reported properties translate to the preparations investigated in the present work. Moreover, the volatility, instability, and hydrophobicity of essential oil constituents may limit their therapeutic applicability, although lipid-based delivery systems could improve their bioavailability [54,92,93].

The volatile profile of the infusion may also contribute, alongside polyphenols, to the documented health-promoting properties of *S. ebulus* preparations. For the first time, the VOCs of an infusion prepared from dried fruits have been characterized. The main compounds included linalool, methyl butanoate, benzyl acetate, and hexahydrofarnesyl acetone. While linalool has been extensively investigated, available information on the other compounds remains limited [94,95,96]. However, methyl butyrate has shown cytotoxic effects in breast cancer cells [97]. Nevertheless, the contribution of these VOCs to the biological effects of *S. ebulus* fruit infusions remains to be elucidated.

Docking studies have previously examined linalool interactions with AChE and MAO, whereas data for the other detected volatiles against AChE, BChE, MAO-A/B, or COX-2 are lacking [98,99]. Our findings extend this knowledge by suggesting that these compounds may interact with multiple biological targets. Hexahydrofarnesyl acetone exhibited the strongest predicted interactions with MAO-A, MAO-B, COX-2, and AChE, while methyl linoleate and methyl oleate demonstrated notable affinity toward MAO isoforms. Benzyl acetate and linalool displayed comparatively weaker predicted binding. Reference inhibitors, including clorgiline, selegiline, donepezil, and celecoxib, showed the expected stronger target-specific interactions, although several VOCs demonstrated comparable docking scores against MAO enzymes. These results should be regarded as preliminary computational predictions requiring further experimental validation.

Bioavailability is crucial for assessing therapeutic potential. Linalool’s poor solubility limits its bioavailability, although oral delivery as part of the whole oil improves absorption compared to the isolated compound, with no plasma accumulation observed in rats [100,101,102,103]. According to Wang et al., its bioavailability is likely slightly higher in humans than in rats [92,97]. Complexation of linalool with β-cyclodextrin or incorporation into nanostructured lipid carriers significantly improves its bioavailability [102,104]. Experimental studies also suggest beneficial gastrointestinal and anti-inflammatory effects in animal models, while blood–brain barrier penetration has so far been demonstrated only in mice [105,106,107,108,109]. For methyl butanoate, hexahydrofarnesyl acetone, and benzyl acetate, pharmacokinetic data remain scarce [110,111]. Therefore, dedicated pharmacokinetic studies are needed to clarify the biological relevance of these volatile constituents.

A limitation of this study is the absence of in vitro, in vivo, or clinical validation of the extracts, which currently restricts definitive conclusions about their pharmacological potential. In addition, the in silico analysis focused on the most abundant volatiles, while a molecular dynamics simulation was performed only for hexahydrofarnesyl acetone binding to MAO-A. Further computational and experimental studies are necessary to validate the predicted multitarget interactions and to clarify the functional relevance of *S. ebulus* fruit volatiles.

## 5. Conclusions

Our results contribute to the still underexplored research on the phytochemical composition of dried *S. ebulus* fruits. Fatty acid esters and hexahydrofarnesyl acetone showed higher relative proportions in the essential oil, whereas linalool predominated in the infusion. It is possible that, in addition to polyphenols, these volatile organic compounds may contribute to the previously reported biological properties of *S. ebulus* infusion, thereby supporting its traditional use as a functional food. However, such assumptions remain speculative. The in silico analyses suggest potential interactions of selected volatiles, particularly hexahydrofarnesyl acetone, with enzymes such as MAO-A/B, COX-2, AChE, and BChE, which are associated with oxidative stress, neuroinflammation, and inflammatory processes in general. Overall, the findings provide a computationally derived framework for future in vitro and in vivo validation, rather than definitive conclusions regarding biological activity.

## Figures and Tables

**Table 1:** Qualitative and quantitative profile of volatile compounds identified in dried fruits of *S. ebulus* and the aqueous infusion prepared from them. Results are presented as mean ± SD.

Peak	RT ^a^ (min.)	RI ^b^	Name	Content in Essential Oil (% of Total FID Peak Area ^c^)	Content in Infusion (% of Total FID Peak Area ^c^)
**1**	7.35	722	Methyl butanoate	3.96 ± 0.95	11.83 ± 1.59
**2**	13.26	1160	Benzyl acetate	n.d	9.72 ± 1.17
**3**	15.14	1097	Linalool	6.41 ± 1.39	51.29 ± 2.60
**4**	24.82	1439	(Z)-β-Famesene	0.80 ± 0.08	3.22 ± 0.75
**5**	24.95	1455	(E)-β-Famesene	0.57 ± 0.05	1.92 ± 0.28
**6**	26.34	1528	(Z)-Nerolidol	0.73 ± 0.07	n.d
**7**	26.41	1556	(E)-Nerolidol	0.45 ± 0.04	n.d
**8**	27.61	1609	*n*-Tetradecanal	0.39 ± 0.04	1.92 ± 0.18
**9**	28.82	1647	Isoamyl geranate	0.87 ± 0.08	1.69 ± 0.24
**10**	33.99	1852	Hexahydrofarnesyl acetone	17.94 ± 1.65	6.63 ± 2.00
**11**	36.7	1920	Methyl hexadecanoate	23.97 ± 2.59	3.82 ± 0.37
**12**	38.32	1990	Ethyl hexadecanoate	3.91 ± 0.30	2.17 ± 0.51
**13**	38.68	2018	(6E,10Z)-Pseudo phytol	2.75 ± 0.26	1.55 ± 0.23
**14**	38.84	2025	(6Z,10E)-Pseudo phytol	3.33 ± 0.31	0.84 ± 0.12
**15**	39.81	2092	Methyl linoleate	13.38 ± 1.24	0.63 ± 0.08
**16**	39.9	2099	Methyl oleate	15.61 ± 1.12	0.56 ± 0.08
**17**	40.33	2123	Methyl octadecanoate	3.52 ± 0.33	0.86 ± 0.12

^a^ RT—retention time; ^b^ RI—retention index; ^c^—content, expressed as percentage of total GC-FID peak area; n.d = no detection.

**Table 2:** IFD scores and MMGBSA ΔG binding free energies of the most abundant compounds in the essential oil and infusion.

No.	Compounds	IFD Glide XP Scores (kcal/mol)					MM-GBSA ΔG Bind. (kcal/mol)				
		MAO-A	MAO-B	AChE	BChE	COX-2	MAO-A	MAO-B	AChE	BChE	COX-2
**1**	Benzyl acetate	−8.32	−7.16	−7.07	−6.45	−6.325	−50.31	−41.38	−37.13	−35.04	−38.72
**2**	Hexahydrofarnesyl acetone	−10.04	−9.50	−8.73	−6.66	−8.628	−67.88	−73.65	−57.84	−50.31	−66.96
**3**	Linalool	−6.34	−6.10	−7.25	−5.68	−6.548	−38.53	−41.72	−38.10	−22.93	−31.47
**4**	Methyl butanoate	−5.09	−4.27	−5.55	−3.89	−3.626	−31.45	−31.12	−32.16	−15.71	−25.58
**5**	Methyl hexadecanoate	−8.08	−9.06	−8.18	−5.80	−8.084	−57.65	−54.49	−49.77	−48.85	−66.37
**6**	Methyl linoleate	−9.63	−9.42	−9.23	−6.66	−9.217	−62.04	−71.21	−57.17	−41.46	−56.94
**7**	Methyl oleate	−9.70	−9.21	−8.70	−7.02	−8.560	−67.36	−69.27	−43.83	−59.10	−53.60
**8**	Clorgyline	−7.28	−8.06	-	-	-	−49.45	−50.97	-	-	-
**9**	Selegiline	−7.91	−8.07	-	-	-	−30.82	−52.11	-	-	-
**10**	Donepezil	-	-	−9.53	−8.09	-	-	-	−91.05	−55.54	-
**11**	Celecoxib	-	-	-	-	−10.64	-	-	-	-	−58.27

## Data Availability

The data supporting the findings of this study are available within the article and its Supplementary Materials.

## Supplementary Materials

The following supporting information can be downloaded at: https://www.mdpi.com/article/10.3390/plants15121765/s1, Figure S1: Total ion chromatograms of samples from essential oil (A) and infusion (B) from dried berries of *S. ebulus*. Numbered peaks correspond to the main volatile constituents listed in Table 1 according to retention time.

## Abbreviations

The following abbreviations are used in this manuscript:

AChE	Acetylcholinesterase
BChE	Butyrylcholinesterase
COX-2	Cyclooxygenase-2
EI	Electron Ionization
GC-FID	Gas Chromatography–Flame Ionization Detection
GC-MS	Gas Chromatography–Mass Spectrometry
IFD	Induced Fit Docking
LRI/LRIs	Linear Retention Index/Indices
MAO-A	Monoamine Oxidase A
MAO-B	Monoamine Oxidase B
MD	Molecular Dynamics
MM-GBSA	Molecular Mechanics Generalized Born Surface Area
NPT	Number of particles, Pressure and Temperature
PDB	Protein Data Bank
RMSD	Root Mean Square Deviation
RMSF	Root Mean Square Fluctuation
ROS	Reactive Oxygen Species
SD	Standard Deviation
VOCs	Volatile Organic Compounds
VSGB	Variable-dielectric Surface Generalized Born

## References

[1] Tasinov O., Kiselova-Kaneva Y., Ivanova D. (2013). *Sambucus ebulus*—From Traditional Medicine to Recent Studies. Scr. Sci. Medica.

[2] Jabbari M., Daneshfard B., Emtiazy M., Khiveh A., Hashempur M.H. (2017). Biological Effects and Clinical Applications of Dwarf Elder (*Sambucus ebulus* L): A Review. J. Evid. Based. Complement. Altern. Med..

[3] Tasinov O., Kiselova-Kaneva Y., Ivanova D. (2013). *Sambucus ebulus* L. Fruit Aqueous Infusion Modulates GCL and GPx4 Gene Expression. Bulg. J. Agric. Sci..

[4] Kiselova-Kaneva Y., Galunska B., Nikolova M., Dincheva I., Badjakov I. (2022). High Resolution LC-MS/MS Characterization of Polyphenolic Composition and Evaluation of Antioxidant Activity of *Sambucus ebulus* Fruit Tea Traditionally Used in Bulgaria as a Functional Food. Food Chem..

[5] Bellanti F., Coda A.R.D., Trecca M.I., Lo Buglio A., Serviddio G., Vendemiale G. (2025). Redox Imbalance in Inflammation: The Interplay of Oxidative and Reductive Stress. Antioxidants.

[6] Cryan J.F., O’riordan K.J., Cowan C.S.M., Sandhu K.V., Bastiaanssen T.F.S., Boehme M., Codagnone M.G., Cussotto S., Fulling C., Golubeva A.V. (2019). The Microbiota-Gut-Brain Axis. Physiol. Rev..

[7] Chen H., Zeng Y., Wang D., Li Y., Xing J., Zeng Y., Liu Z., Zhou X., Fan H. (2024). Neuroinflammation of Microglial Regulation in Alzheimer’s Disease: Therapeutic Approaches. Molecules.

[8] Buhrmester R.A., Ebinger J.E., Seigler D.S. (2000). Sambunigrin and Cyanogenic Variability in Populations of *Sambucus canadensis* L. (Caprifoliaceae). Biochem. Syst. Ecol..

[9] Shokrzadeh M., Saeedi Saravi S.S. (2010). The Chemistry, Pharmacology and Clinical Properties of *Sambucus ebulus*: A Review. J. Med. Plants Res..

[10] Mikulic-Petkovsek M., Ivancic A., Todorovic B., Veberic R., Stampar F. (2015). Fruit Phenolic Composition of Different Elderberry Species and Hybrids. J. Food Sci..

[11] Vankova D.V., Todorova M.N., Kisselova-Kaneva Y.D., Galunska B.T. (2019). Development of New and Robust LC-MS Method for Simultaneous Quantification of Polyphenols from *Sambucus ebulus* Fruits. J. Liq. Chromatogr. Relat. Technol..

[12] Tasinov O., Dincheva I., Badjakov I., Kiselova-Kaneva Y., Galunska B., Nogueiras R., Ivanova D. (2021). Phytochemical Composition, Anti-Inflammatory and ER Stress-Reducing Potential of *Sambucus ebulus* L. Fruit Extract. Plants.

[13] Kiselova-Kaneva Y., Nashar M., Roussev B., Salim A., Hristova M., Olczyk P., Komosinska-Vassev K., Dincheva I., Badjakov I., Galunska B. (2023). *Sambucus ebulus* (Elderberry) Fruits Modulate Inflammation and Complement System Activity in Humans. Int. J. Mol. Sci..

[14] Barak T.H., Celep E., İnan Y., Yeşilada E. (2020). In Vitro Human Digestion Simulation of the Bioavailability and Antioxidant Activity of Phenolics from *Sambucus ebulus* L. Fruit Extracts. Food Biosci..

[15] Tasinov O., Kiselova-Kaneva Y., Ivanova D. (2020). Effects of Dwarf Elder Fruit Infusion on Nuclear Factor Kappa B and Glutathione Metabolism-Related Genes Transcription in a Model of Lipopolysaccharides Challenged Macrophages. Bulg. Chem. Commun..

[16] Ivanova D., Tasinov O., Kiselova-Kaneva Y. (2014). Improved Lipid Profile and Increased Serum Antioxidant Capacity in Healthy Volunteers after *Sambucus ebulus* L. Fruit Infusion Consumption. Int. J. Food Sci. Nutr..

[17] Stoyanov S., Barbolov M., Yaneva G., Tasinov O. (2025). Modulation of Endoplasmic Reticulum Stress by Selected Polyphenols from *Sambucus ebulus* L. Fruit. Plants.

[18] Ahmad A., Elisha I.L., van Vuuren S., Viljoen A. (2021). Volatile Phenolics: A Comprehensive Review of the Anti-Infective Properties of an Important Class of Essential Oil Constituents. Phytochemistry.

[19] Mohamed A.A., Alotaibi B.M. (2022). Essential Oils of Some Medicinal Plants and Their Biological Activities: A Mini Review. J. Umm Al-Qura Univ. Appl. Sci..

[20] Masyita A., Mustika Sari R., Dwi Astuti A., Yasir B., Rahma Rumata N., Emran T.B., Nainu F., Simal-Gandara J. (2022). Terpenes and Terpenoids as Main Bioactive Compounds of Essential Oils, Their Roles in Human Health and Potential Application as Natural Food Preservatives. Food Chem. X.

[21] Rout S., Tambe S., Deshmukh R.K., Mali S., Cruz J., Srivastav P.P., Amin P.D., Gaikwad K.K., Andrade E.H.d.A., de Oliveira M.S. (2022). Recent Trends in the Application of Essential Oils: The next Generation of Food Preservation and Food Packaging. Trends Food Sci. Technol..

[22] Santana de Oliveira M., Vostinaru O., Rigano D., de Aguiar Andrade E.H. (2023). Editorial: Bioactive Compounds Present in Essential Oils: Advances and Pharmacological Applications. Front. Pharmacol..

[23] Sytykiewicz H., Goławska S., Łukasik I. (2025). New Insights into the Synergistic Bioactivities of *Zingiber officinale* (Rosc.) and *Humulus lupulus* (L.) Essential Oils: Targeting Tyrosinase Inhibition and Antioxidant Mechanisms. Molecules.

[24] Feizbakhsh A., Pazoki H., Ebrahimzadeh M.A. (2010). *Sambucus ebulus*, Introduction to Mechanism of Action; A Chemical Viewpoint. Pharmacologyonline.

[25] Feizbakhsh A., Pazoki H., Ebrahimzadeh M.A. (2016). Effect of Gibberellic Acid on Composition of *S. ebulus* Leaf Essential Oil (*Caprifoliaceous*). Pharmacologyonline.

[26] Feizbakhsh A., Pazoki H., Mohammadrezaei V., Ebrahimzadeh M. (2014). Effect of Phytohormones on the Composition of *Sambucus ebulus* Leaf Essential Oil. Trop. J. Pharm. Res..

[27] Pribela A., Durcanska J., Piry J., Karovicova J. (1992). Volatile Substances of Dwarf Elder (*Sambucus ebulus*) Fruits. Biologia. Ser. C..

[28] Kurban B., Osmaniye D., Sağlık Özkan B.N., Kaplancıklı Z.A. (2024). Investigation of Dual AChE/MAO Inhibitory Activities of New Morpholine and Piperazine Structured Compounds. Eur. J. Life Sci..

[29] Farooq U., Islam M., Batool Z., Mali S.N., Jawarkar R.D., Gurav S.S., Alharthy R.D., Şenol H., Sadeghian N., Taslimi P. (2025). Design, Synthesis, In Vitro, and In Silico Studies of 5-(Diethylamino)-2-Formylphenyl Naphthalene-2-Sulfonate Based Thiosemicarbazones as Potent Anti-Alzheimer Agents. Arch. Pharm..

[30] García-Domínguez M. (2025). Neuroinflammation: Mechanisms, Dual Roles, and Therapeutic Strategies in Neurological Disorders. Curr. Issues Mol. Biol..

[31] Asghar S., Mushtaq N., Ahmed A., Anwar L., Munawar R., Akhtar S. (2024). Potential of Tryptamine Derivatives as Multi-Target Directed Ligands for Alzheimer’s Disease: AChE, MAO-B, and COX-2 as Molecular Targets. Molecules.

[32] Moussa N., Dayoub N. (2023). Exploring the Role of COX-2 in Alzheimer’s Disease: Potential Therapeutic Implications of COX-2 Inhibitors. Saudi Pharm. J..

[33] Şenol H., Çağman Z., Gençoğlu Katmerlikaya T., Sinan Tokalı F. (2023). New Anthranilic Acid Hydrazones as Fenamate Isosteres: Synthesis, Characterization, Molecular Docking, Dynamics & in Silico ADME, in Vitro Anti-Inflammatory and Anticancer Activity Studies. Chem. Biodivers..

[34] Behl T., Kaur D., Sehgal A., Singh S., Sharma N., Zengin G., Andronie-Cioara F.L., Toma M.M., Bungau S., Bumbu A.G. (2021). Role of Monoamine Oxidase Activity in Alzheimer’s Disease: An Insight into the Therapeutic Potential of Inhibitors. Molecules.

[35] Banik A., Amaradhi R., Lee D., Sau M., Wang W., Dingledine R., Ganesh T. (2021). Prostaglandin EP2 Receptor Antagonist Ameliorates Neuroinflammation in a Two-Hit Mouse Model of Alzheimer’s Disease. J. Neuroinflamm..

[36] Demir Y., Şenol H., Uluçay O., Ateşoğlu Ş., Tokalı F.S. (2026). Morpholine-Modified Thiosemicarbazones and Thiazolidin-4-Ones against Alzheimer’s Key Enzymes: From Synthesis to Inhibition. Comput. Biol. Chem..

[37] Sadeghi M., Karami M., Miroliaei M., Ghanadian M. (2025). In Silico Approaches for Rational Drug Design and Potential Enzyme Inhibitors Discovery: A Mini-Review. J. Adv. Biomed. Sci..

[38] Ulker O.C., Ulker O., Hiziroglu S. (2021). Volatile Organic Compounds (VOCs) Emitted from Coated Furniture Units. Coatings.

[39] Toraman G.Ö.A., Atasoy S., Şenol H., Şükran Okudan E., Öykü Dinç H., Topçu G. (2024). LC–MS and GC-MS Analyses on Green Algae Penicillus Capitatus: Cytotoxic, Antimicrobial and Anticholinesterase Activity Screening Enhanced by Molecular Docking & Dynamics and ADME Studies. Chem. Biodivers..

[40] Naz M., Şenol H., Okudan E.Ş., Sayın S., Konuklugil B., Topçu G. (2024). Analyzing Eight Turkish Macroalgae: Fatty Acids, Proteins, and in Silico Biological Activity Profiles. Eur. J. Lipid Sci. Technol..

[41] Senol H., Ozgun-Acar O., Dağ A., Eken A., Guner H., Aykut Z.G., Topcu G., Sen A. (2023). Synthesis and Comprehensive in Vivo Activity Profiling of Olean-12-En-28-Ol, 3β-Pentacosanoate in Experimental Autoimmune Encephalomyelitis: A Natural Remyelinating and Anti-Inflammatory Agent. J. Nat. Prod..

[42] Dimitrova D., Kehayova G., Dimitrova S., Dragomanova S. (2025). Marine-Derived Natural Substances with Anticholinesterase Activity. Mar. Drugs.

[43] Ju Z., Li M., Xu J., Howell D.C., Li Z., Chen F.-E. (2022). Recent Development on COX-2 Inhibitors as Promising Anti-Inflammatory Agents: The Past 10 Years. Acta Pharm. Sin. B.

[44] Chaurasiya N., Leon F., Muhammad I., Tekwani B. (2022). Natural Products Inhibitors of Monoamine Oxidases—Potential New Drug Leads for Neuroprotection, Neurological Disorders, and Neuroblastoma. Molecules.

[45] Arfeen M., Dhaked D.K., Mani V. (2025). Multipotent Effect of Clozapine on Lipopolysaccharide-Induced Acetylcholinesterase, Cyclooxygenase-2,5-Lipoxygenase, and Caspase-3: In Vivo and Molecular Modeling Studies. Molecules.

[46] Mirza F.J., Zahid S., Amber S., Sumera S., Jabeen H., Asim N., Ali Shah S.A. (2022). Multitargeted Molecular Docking and Dynamic Simulation Studies of Bioactive Compounds from Rosmarinus Officinalis against Alzheimer’s Disease. Molecules.

[47] Tao G., Irie Y., Li D.J., Wing M.K. (2005). Eugenol and Its Structural Analogs Inhibit Monoamine Oxidase A and Exhibit Antidepressant-like Activity. Bioorg. Med. Chem..

[48] Guo Z.-H., Huang J.-Y., Xiao T., Yang W. (2023). Terpenoids as Anti-Inflammatory Substances Inhibiting COX-2 Isolated from the Fibrous Roots of *Alangium chinense* (Lour.) Harms. Nat. Prod. Res..

[49] Li J.-Y., Dong S.-H., Zhang X., Liu Z.-J., Hao J.-L., Lin B., Bai M., Huang X.-X., Song S.-J. (2023). Structurally Diverse Terpenoids from *Elephantopus scaber* L. and Their Acetylcholinesterase Inhibitory Activities. Phytochemistry.

[50] Tokalı F.S., Şenol H., Ateşoğlu Ş., Çakır F., Tokalı P., Akbaş F. (2026). Design and Synthesis of New Thienopyrimidine Derivatives as Potential Anticancer Agents: From Cytotoxicity Screening to VEGFR Inhibition Modeling. J. Mol. Struct..

[51] Tokalı F.S., Şenol H., Ateşoğlu Ş., Tokalı P., Akbaş F. (2025). Exploring Highly Selective Polymethoxy Fenamate Isosteres as Novel Anti-Prostate Cancer Agents: Synthesis, Biological Activity, Molecular Docking, Molecular Dynamics, and ADME Studies. J. Mol. Struct..

[52] Papagrigoriou T., Iliadi P., Mitić M.N., Mrmošanin J.M., Papanastasi K., Karapatzak E., Maloupa E., Gkourogianni A.V., Badeka A.V., Krigas N. (2023). Wild-Growing and Conventionally or Organically Cultivated Sambucus Nigra Germplasm: Fruit Phytochemical Profile, Total Phenolic Content, Antioxidant Activity, and Leaf Elements. Plants.

[53] Najar B., Ferri B., Cioni P.L., Pistelli L. (2021). Volatile Emission and Essential Oil Composition of *Sambucus nigra* L. Organs during Different Developmental Stages. Plant Biosyst.—Int. J. Deal. All Asp. Plant Biol..

[54] Cimino C., Maurel O.M., Musumeci T., Bonaccorso A., Drago F., Souto E.M.B., Pignatello R., Carbone C. (2021). Essential Oils: Pharmaceutical Applications and Encapsulation Strategies into Lipid-Based Delivery Systems. Pharmaceutics.

[55] Zengin G., Mollica A., Arsenijević J., Pavlić B., Zeković Z., Sinan K.I., Yan L., Cvetanović Kljakić A., Ražić S. (2022). A Comparative Study of Chamomile Essential Oils and Lipophilic Extracts Obtained by Conventional and Greener Extraction Techniques: Chemometric Approach to Chemical Composition and Biological Activity. Separations.

[56] Chan W.K., Tan L.T.H., Chan K.G., Lee L.H., Goh B.H. (2016). Nerolidol: A Sesquiterpene Alcohol with Multi-Faceted Pharmacological and Biological Activities. Molecules.

[57] Yan Y., Zhang Y., Zhang B., Yang C., Zhang F., Wei C., Zhou X., Zhu X., Li X. (2025). Topical Exposure to (E)-β-Farnesene Alters Behavior, Reproduction, and Wing Dimorphism in the English Grain Aphid, Sitobion Avenae (Hemiptera: Aphididae). J. Econ. Entomol..

[58] Gawde A., Cantrell C.L., Zheljazkov V.D., Astatkie T., Schlegel V. (2014). Steam Distillation Extraction Kinetics Regression Models to Predict Essential Oil Yield, Composition, and Bioactivity of Chamomile Oil. Ind. Crops Prod..

[59] Meenongyai W., Kaewka K., Wongpanit K., Phongkaew P., Khejornsart P., Khumpeerawat P., Stelzleni A.M. (2025). Aging Time Influences Fatty Acid Profiles and Volatile Compounds in Cooked Thai Native Beef. J. Adv. Vet. Anim. Res..

[60] Zahmanov G., Alipieva K., Denev P., Todorov D., Hinkov A., Shishkov S., Simova S., Georgiev M.I. (2015). Flavonoid Glycosides Profiling in Dwarf Elder Fruits (*Sambucus ebulus* L.) and Evaluation of Their Antioxidant and Anti-Herpes Simplex Activities. Ind. Crops Prod..

[61] Luo Z., Murray B.S., Yusoff A., Morgan M.R.A., Povey M.J.W., Day A.J. (2011). Particle-Stabilizing Effects of Flavonoids at the Oil−Water Interface. J. Agric. Food Chem..

[62] Díaz-Reinoso B., Rivas S., Rivas J., Domínguez H. (2023). Subcritical Water Extraction of Essential Oils and Plant Oils. Sustain. Chem. Pharm..

[63] Joo C., Noh H.-K. (2020). Engine Performance of Ethyl Butyrate and Methyl Butyrate Blended Gasoline. J. Energy Eng..

[64] Masango P. (2005). Cleaner Production of Essential Oils by Steam Distillation. J. Clean. Prod..

[65] Pereira I., Severino P., Santos A.C., Silva A.M., Souto E.B. (2018). Linalool Bioactive Properties and Potential Applicability in Drug Delivery Systems. Colloids Surf. B Biointerfaces.

[66] Meyyappan N., Nagendra Gandhi N. (2004). Solubility and Mass Transfer Coefficient Enhancement of Benzyl Acetate in Water through Hydrotropy. J. Chem. Eng. Data.

[67] Tirillini B., Maggi F. (2021). Volatile Organic Compounds of the Glandular Trichomes of *Ocimum basilicum* and Artifacts during the Distillation of the Leaves. Appl. Sci..

[68] Seol G.-H., Kang P., Lee H.S., Seol G.H. (2016). Antioxidant Activity of Linalool in Patients with Carpal Tunnel Syndrome. BMC Neurol..

[69] Wu Q., Yu L., Qiu J., Shen B., Wang D., Soromou L.W., Feng H. (2014). Linalool Attenuates Lung Inflammation Induced by Pasteurella Multocida via Activating Nrf-2 Signaling Pathway. Int. Immunopharmacol..

[70] Katsuyama S., Kuwahata H., Yagi T., Kishikawa Y., Komatsu T., Sakurada T., Nakamura H. (2012). Intraplantar Injection of Linalool Reduces Paclitaxel-Induced Acute Pain in Mice. Biomed. Res..

[71] Dias I.J., Trajano E.R.I.S., Castro R.D., Ferreira G.L.S., Medeiros H.C.M., Gomes D.Q.C. (2017). Antifungal Activity of Linalool in Cases of Candida Spp. Isolated from Individuals with Oral Candidiasis. Braz. J. Biol..

[72] Liu X., Cai J., Chen H., Zhong Q., Hou Y., Chen W., Chen W. (2020). Antibacterial Activity and Mechanism of Linalool against Pseudomonas Aeruginosa. Microb. Pathog..

[73] Park S.-N., Lim Y.K., Freire M.O., Cho E., Jin D., Kook J.-K. (2012). Antimicrobial Effect of Linalool and α-Terpineol against Periodontopathic and Cariogenic Bacteria. Anaerobe.

[74] Chang M.-Y., Shieh D.-E., Chen C.-C., Yeh C.-S., Dong H.-P. (2015). Linalool Induces Cell Cycle Arrest and Apoptosis in Leukemia Cells and Cervical Cancer Cells through CDKIs. Int. J. Mol. Sci..

[75] Villamizar L.H., Cardoso M.d.G., de Andrade J., Teixeira M.L., Soares M.J. (2017). Linalool, a Piper Aduncum Essential Oil Component, Has Selective Activity against Trypanosoma Cruzi Trypomastigote Forms at 4 °C. Mem. Inst. Oswaldo Cruz.

[76] Camargo S.B., Simões L.O., Medeiros C.F.d.A., de Melo Jesus A., Fregoneze J.B., Evangelista A., Villarreal C.F., Araújo A.A.d.S., Quintans-Júnior L.J., Silva D.F. (2018). Antihypertensive Potential of Linalool and Linalool Complexed with β-Cyclodextrin: Effects of Subchronic Treatment on Blood Pressure and Vascular Reactivity. Biochem. Pharmacol..

[77] Mohamed M.E., Abduldaium M.S., Younis N.S. (2021). Cardioprotective Effect of Linalool against Isoproterenol-Induced Myocardial Infarction. Life.

[78] da Silva F.V., de Barros Fernandes H., Oliveira I.S., Viana A.F.S.C., da Costa D.S., Lopes M.T.P., de Lira K.L., Quintans-Júnior L.J., de Sousa A.A., de Cássia Meneses Oliveira R. (2016). Beta-Cyclodextrin Enhanced Gastroprotective Effect of (−)-Linalool, a Monoterpene Present in Rosewood Essential Oil, in Gastric Lesion Models. Naunyn. Schmiedebergs Arch. Pharmacol..

[79] Abdel Jaleel G.A., Azab S.S., El-Bakly W.M., Hassan A. (2021). Methyl Palmitate Attenuates Adjuvant Induced Arthritis in Rats by Decrease of CD68 Synovial Macrophages. Biomed. Pharmacother..

[80] Saeed N.M., El-Demerdash E., Abdel-Rahman H.M., Algandaby M.M., Al-Abbasi F.A., Abdel-Naim A.B. (2012). Anti-Inflammatory Activity of Methyl Palmitate and Ethyl Palmitate in Different Experimental Rat Models. Toxicol. Appl. Pharmacol..

[81] Lee Y.-C., Chang H.-H., Chiang C.-L., Liu C.-H., Yeh J.-I., Chen M.-F., Chen P.-Y., Kuo J.-S., Lee T.J.F. (2011). Role of Perivascular Adipose Tissue–Derived Methyl Palmitate in Vascular Tone Regulation and Pathogenesis of Hypertension. Circulation.

[82] Lee Y.-C., Chang H.-H., Liu C.-H., Chen M.-F., Chen P.-Y., Kuo J.-S., Lee T.J.-F. (2010). Methyl Palmitate: A Potent Vasodilator Released in the Retina. Investig. Ophthalmol. Vis. Sci..

[83] Ayoub I.M., Korinek M., El-Shazly M., Wetterauer B., El-Beshbishy H.A., Hwang T.L., Chen B.H., Chang F.R., Wink M., Singab A.N.B. (2021). Anti-Allergic, Anti-Inflammatory, and Anti-Hyperglycemic Activity of Chasmanthe Aethiopica Leaf Extract and Its Profiling Using LC/MS and GLC/MS. Plants.

[84] Avoseh O.N., Mtunzi F.M., Ogunwande I.A., Ascrizzi R., Guido F. (2021). Albizia Lebbeck and Albizia Zygia Volatile Oils Exhibit Anti-Nociceptive and Anti-Inflammatory Properties in Pain Models. J. Ethnopharmacol..

[85] Szewczyk K., Kalemba D., Komsta Ł., Nowak R. (2016). Comparison of the Essential Oil Composition of Selected Impatiens Species and Its Antioxidant Activities. Molecules.

[86] Radulović N., Stojanović G., Palić R. (2006). Composition and Antimicrobial Activity of *Equisetum arvense* L. Essential Oil. Phytother. Res..

[87] He J., Xiong W., Zhao L., Liu B., Huang Y. (2023). Anti-α-Glucosidase, Anti-Proliferative and Anti-Enterovirus 71 Activity of Secondary Metabolites Identified from *Grifola frondosa*. Plant Foods Hum. Nutr..

[88] Ko G.-A., Shrestha S., Kim Cho S. (2018). Sageretia Thea Fruit Extracts Rich in Methyl Linoleate and Methyl Linolenate Downregulate Melanogenesis via the Akt/GSK3β Signaling Pathway. Nutr. Res. Pract..

[89] Yoon Y.P., Ryu J., Park S.H., Lee H.J., Lee S., Lee S.K., Kim J.-O., Hong J.-H., Seok J.H., Lee C.J. (2014). Effects of Lobetyolin, Lobetyol and Methyl Linoleate on Secretion, Production and Gene Expression of MUC5AC Mucin from Airway Epithelial Cells. Tuberc. Respir. Dis..

[90] Pinto M.E.A., Araujo S.G., Morais M.I., Sa N.P., Lima C.M., Rosa C.A., Siqueira E.P., Johann S., Lima L.A.R.S. (2017). Antifungal and Antioxidant Activity of Fatty Acid Methyl Esters from Vegetable Oils. An. Acad. Bras. Cienc..

[91] Padmini N., Rashiya N., Sivakumar N., Kannan N.D., Manjuladevi R., Rajasekar P., Prabhu N.M., Selvakumar G. (2020). In Vitro and in Vivo Efficacy of Methyl Oleate and Palmitic Acid against ESBL Producing MDR Escherichia Coli and Klebsiella Pneumoniae. Microb. Pathog..

[92] Wang Y.-H., Mondal G., Stevens N., Bascoul C., Osguthorpe R.J., Khan I.A., Yates C.R. (2023). Development of a Liquid Chromatography–Tandem Mass Spectrometry (LC–MS/MS) Method for Characterizing Linalool Oral Pharmacokinetics in Humans. Molecules.

[93] Hematizadeh A., Ebrahimzadeh M.A., Sarvi S., Sadeghi M., Daryani A., Gholami S., Nayeri T., Hosseini S.A. (2023). In Vitro and In Vivo Anti-Parasitic Activity of *Sambucus ebulus* and Feijoa Sellowiana Extracts Silver Nanoparticles on Toxoplasma Gondii Tachyzoites. Acta Parasitol..

[94] Muhammad U., Ullah R., Subhan Z., Bahadar H., Ahmad S., Rasheed A. (2024). Evaluation of In-Vivo Anti-Inflammatory Activity of Methyl 2-(5-Butyl-6-Thioxo-1, 3, 5-Thiadiazinan-3yl), Butanoate: In-Vivo Anti-Inflammatory Activity of Methyl Thiadiazinan-3yl Butanoate. Pak. J. Health Sci..

[95] Sun Y., Ran Y., Yang H., Mo M., Li G. (2023). Volatile Metabolites from Brevundimonas Diminuta and Nematicidal Esters Inhibit Meloidogyne Javanica. Microorganisms.

[96] Lachenmeier W., Zieniuk B., Jasí Nska K., Wierzchowska K., Fabiszewska A. (2022). Enzymatic Synthesis of Flavours and Fragrances, Antioxidants and Antimicrobials on the Example of Benzyl Alcohol and Its Selected Derivatives. Biol. Life Sci. Forum.

[97] Khan M.A., Ahmad R., Srivastava A.N. (2016). Effect of Methyl Butyrate Aroma on the Survival and Viability of Human Breast Cancer Cells in Vitro. J. Egypt. Natl. Canc. Inst..

[98] Dhananjayan K., Sumathy A., Palanisamy S. (2013). Molecular Docking Studies and In-Vitro Acetylcholinesterase Inhibition by Terpenoids and Flavonoids. Asian J. Res. Chem..

[99] Rayff Da Silva P., Cabral De Andrade J., Ferreira De Sousa N., Ribeiro Portela A.C., Fernandes H., Pires O., Rodrigues M.C., Remígio B., Da D., Alves N. (2023). Computational Studies Applied to Linalool and Citronellal Derivatives Against Alzheimer’s and Parkinson’s Disorders: A Review with Experimental Approach. Curr. Neuropharmacol..

[100] Nöldner M., Germer S., Koch E. (2011). Pharmacokinetics of Linalool and Linalyl Acetate, the Two Main Constituents of Silexan, an Essential Oil from Lavandula Angustifolia Flowers, in Rats. Planta Med..

[101] Nöldner M., Germer S., Koch E. (2013). Pharmacokinetics of Linalool and Linalyl Acetate in Rats after Repeated Oral Administration of Silexan, an Essential Oil from Lavandula Angustifolia Flowers. Planta Med..

[102] Shi F., Zhao Y., Firempong C.K., Xu X. (2016). Preparation, Characterization and Pharmacokinetic Studies of Linalool-Loaded Nanostructured Lipid Carriers. Pharm. Biol..

[103] Mondal G., Dale O.R., Wang Y.-H., Khan S.I., Khan I.A., Yates C.R. (2023). In Vitro Metabolism and CYP-Modulating Activity of Lavender Oil and Its Major Constituents. Molecules.

[104] Pedreira J.C.G., Filho E.R.d.O., Pereira H.P.O.C., Martins A.F.C., Schoepfer F.D., Nogueira R.R.G., de Oliveira J.D., Pimentel H.J.d.S., Mendonça J.A., Costa P.O.M. (2024). Cardiovascular Effects Of Free Or Complexed Linalool With Β-Cyclodextrin: A Focus For Antihypertensive Action. Braz. J. Implantol. Health Sci..

[105] Iwasaki K., Zheng Y.W., Murata S., Ito H., Nakayama K., Kurokawa T., Sano N., Nowatari T., Villareal M.O., Nagano Y.N. (2016). Anticancer Effect of Linalool via Cancer-Specific Hydroxyl Radical Generation in Human Colon Cancer. World J. Gastroenterol..

[106] Tan N., Zhao M., Luo Z., Li Z., Zhang X., Xu J., Gu X., Wang Q., Ding S., Ying M. (2024). Linalool as a Key Component in Strawberry Volatile Organic Compounds (VOCs) Modulates Gut Microbiota, Systemic Inflammation, and Glucolipid Metabolism. Food Chem..

[107] Periyasamy T., Sathibabu Uddandrao V.V., Ponnusamy C., Ganapathy S., Pudhupalayam S.P., Singaravel S., Ponnusamy P., Ramasamy J., Aiyasamy K., Sasikumar V. (2023). Linalool Mitigated High-Fat Diet–Induced Non-Alcoholic Fatty Liver Disease by Regulating the Intestinal-Hepatic Axis via TGF-β/NF-KB/TLR4/ZO-1 Pathway. Rev. Bras. Farmacogn..

[108] Barocelli E., Calcina F., Chiavarini M., Impicciatore M., Bruni R., Bianchi A., Ballabeni V. (2004). Antinociceptive and Gastroprotective Effects of Inhaled and Orally Administered Lavandula Hybrida Reverchon “Grosso” Essential Oil. Life Sci..

[109] Umezu T., Sano T., Hayashi J., Shibata Y. (2020). Simultaneous Blood and Brain Microdialysis in a Free-Moving Mouse to Test Blood-Brain Barrier Permeability of Chemicals. Toxicol. Rep..

[110] Zhou G., Zhou G. (2024). Exploring Ester Prodrugs: A Comprehensive Review of Approaches, Applications, and Methods. Pharmacol. Pharm..

[111] Abdo K.M., Huff J.E., Haseman J.K., Boorman G.A., Eustis S.L., Matthews H.B., Burka L.T., Prejean J.D., Thompson R.B. (1985). Benzyl Acetate Carcinogenicity, Metabolism, and Disposition in Fischer 344 Rats and B6C3F1 Mice. Toxicology.

