# Supplementary material for: Comparative Profiling and In Silico Multitarget Analysis of Volatile Constituents from Sambucus ebulus L. Dried Fruits
Source: Plants (Basel). 2026 Jun 8;15(12):1765. doi: 10.3390/plants15121765 (PMC13306616; doi:10.3390/plants15121765)
Supplement: Supplementary file 1 [file plants-15-01765-s001.zip › plants-4279744-supplementary.pdf]

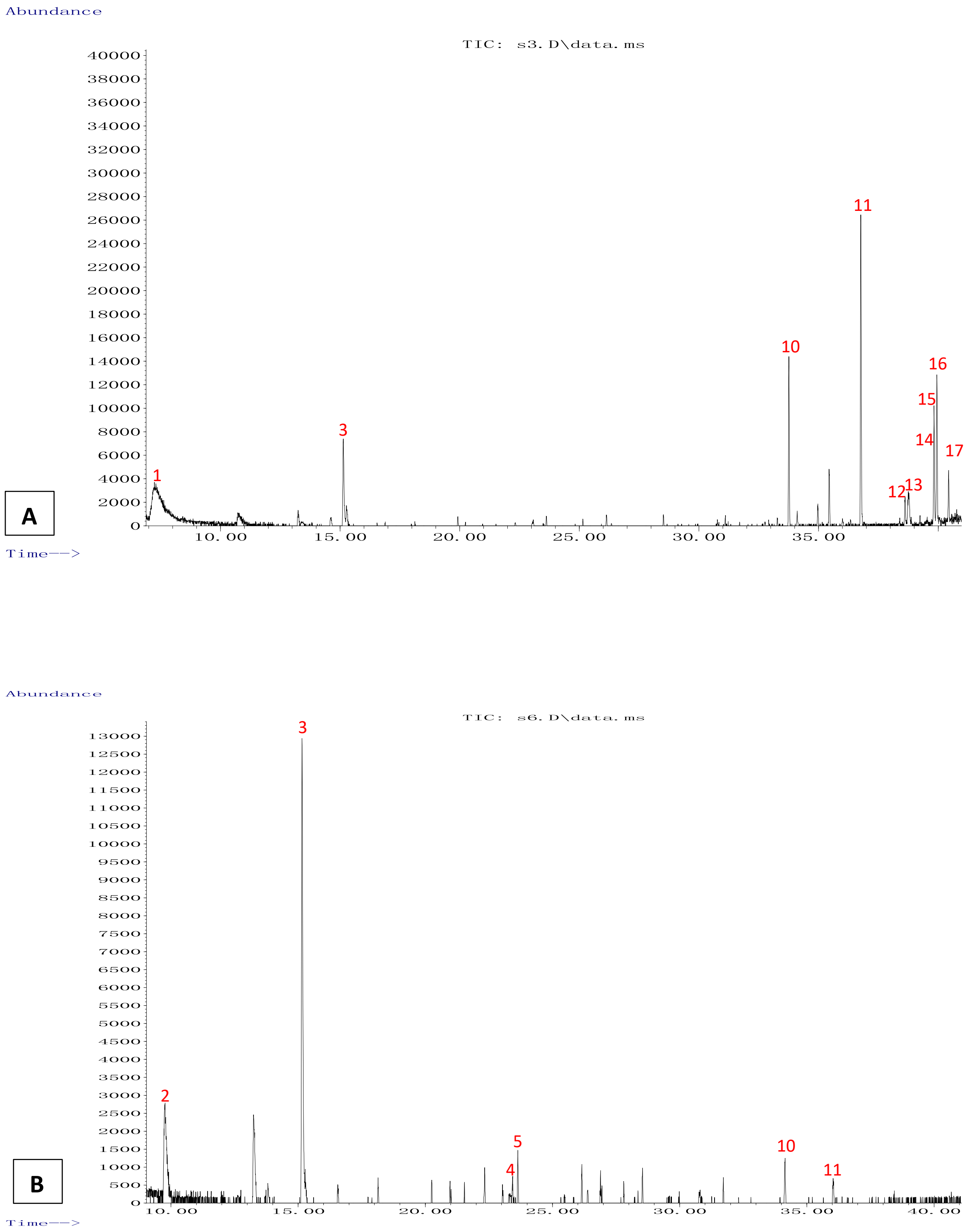

Figure S1. Total ion chromatograms of samples from essential oil **(A)** and infusion **(B)** from dried berries of *S. ebulus.* Numbered peaks correspond to the main volatile constituents listed in Table 1 according to retention time.
